# Initial Experience with Standalone Thoracoscopic Left Atrial Appendage Occlusion Using AtriClip Pro2: A Single-Center Retrospective Cohort Study in Latvia

**DOI:** 10.3390/medicina62091786

**Published:** 2026-09-16

**Authors:** Diāna Kalniņa, Kaspars Kupics, Renāta Rimdjonoka, Inga Urtāne, Daniels Siļčonoks, Andrejs Ērglis, Pēteris Stradiņš

**Affiliations:** 1 Pauls Stradins Clinical University Hospital, LV-1002 Riga, Latvia; 2 Faculty of Medicine and Life Sciences, University of Latvia, LV-1004 Riga, Latvia; 3 Faculty of Medicine, Riga Stradins University, LV-1007 Riga, Latvia; 4 Department of Pharmacology and Pharmacotherapy, Faculty of Pharmacy, Riga Stradins University, LV-1007 Riga, Latvia

**Keywords:** atrial fibrillation, minimally invasive cardiac surgery, thoracoscopic LAA occlusion, AtriClip, oral anticoagulation contraindications, stroke prevention

## Abstract

*Background and Objectives:* Left atrial appendage (LAA) closure is an established strategy for stroke prevention in selected patients with atrial fibrillation (AF), particularly when long-term oral anticoagulation (OAC) is contraindicated. Thoracoscopic epicardial LAA occlusion using the AtriClip Pro2 device provides a minimally invasive surgical approach; however, evidence for standalone use remains limited. This study aimed to describe the initial feasibility and early clinical outcomes of standalone thoracoscopic LAA occlusion using AtriClip Pro2 in Latvia. *Materials and Methods:* We performed a single-center retrospective observational study including all consecutive patients who underwent standalone thoracoscopic LAA occlusion at the Department of Cardiac Surgery, Pauls Stradins Clinical University Hospital, between December 2024 and March 2026. Baseline characteristics, procedural outcomes, perioperative complications, rehospitalizations, and antithrombotic therapy status were assessed. LAA closure was assessed by transesophageal echocardiography (TEE). *Results:* Fourteen patients were included. Mean age was 63.6 ± 16.7 years (median 67.0; IQR 54.8–76.8), and 64.3% were female. Thoracoscopic completion was achieved in 13/14 patients (92.9%; 95% CI 68.5–98.7%); one patient (7.1%) required conversion to median sternotomy because of intraoperative bleeding. Complete LAA exclusion without residual flow or stump was documented in all 14 patients (100%; 95% CI 78.5–100%). No perioperative stroke or mortality occurred. At three months, follow-up data were available for all patients, and antithrombotic therapy had been discontinued in 12/14 patients (85.7%). *Conclusions:* This small, heterogeneous, single-center series supports the technical feasibility of standalone thoracoscopic AtriClip Pro2 implantation in selected patients. These encouraging early findings warrant further evaluation in larger cohorts with longer clinical follow-up and standardized postoperative imaging.

## 1. Introduction

Atrial fibrillation (AF) is the most common sustained cardiac arrhythmia and is associated with a substantially increased risk of ischemic stroke, heart failure, and mortality. According to recent estimates, AF affects approximately 2–3% of the adult population, and its prevalence is expected to increase further because of population aging and improved survival of patients with cardiovascular disease [1,2]. According to data from the Centre for Disease Prevention and Control (SPKC), mortality in the diagnostic group ‘atrial fibrillation and flutter’ increased by 208% between 2015 and 2022 [3].

In patients with non-valvular AF, more than 90% of cardiac thrombi originate from the left atrial appendage (LAA), making this structure an important target for stroke prevention strategies [4,5]. Long-term oral anticoagulation (OAC) remains the standard treatment for reducing thromboembolic risk. However, a substantial proportion of patients have contraindications to anticoagulation because of previous major bleeding, intracranial hemorrhage, recurrent gastrointestinal bleeding, or other conditions associated with an increased hemorrhagic risk. In addition, thromboembolic events may occur despite adequate anticoagulation therapy [1,6].

LAA occlusion has emerged as an alternative strategy for stroke prevention in selected patients who are unsuitable for long-term OAC [7]. Another potential indication for thoracoscopic LAA closure is the management of atrial tachycardia or atrial fibrillation originating from the LAA in patients who have undergone unsuccessful endocardial catheter ablation [8].

Transcatheter devices, including Watchman and Amulet, are widely used and have demonstrated favorable clinical outcomes in appropriately selected patients. More recently, thoracoscopic epicardial LAA closure using the AtriClip device has gained increasing interest as a minimally invasive surgical alternative. Unlike transcatheter approaches, epicardial clipping excludes the appendage without leaving an intracardiac implant and may facilitate discontinuation of antithrombotic therapy following confirmation of complete closure [7,9].

Previous studies have demonstrated high procedural success rates and durable LAA exclusion following thoracoscopic AtriClip PRO2 implantation, with low rates of perioperative complications and thromboembolic events during follow-up [7]. Potential complications of thoracoscopic AtriClip implantation reported in the literature include cardiac tamponade, left atrial perforation, incomplete LAA closure with a residual stump, postoperative thoracic bleeding, and access-site bleeding. Published studies have reported relatively low rates of these complications [10,11]. Nevertheless, published experience remains limited compared with transcatheter techniques, and data from the Baltic region are lacking.

The aim of this study was to evaluate the initial clinical experience and early outcomes of standalone thoracoscopic LAA occlusion using the AtriClip Pro2 device at the national cardiac surgery center in Latvia.

## 2. Materials and Methods

### 2.1. Study Design

This was a single-center retrospective observational cohort study conducted at the Department of Cardiac Surgery, Pauls Stradins Clinical University Hospital, Riga, Latvia. All consecutive patients who underwent standalone LAA occlusion using the AtriClip Pro2 device between December 2024 and 31 March 2026 were included in the study. Clinical follow-up continued beyond the procedural inclusion period, and the database was closed on 1 July 2026, ensuring a minimum follow-up of three months for all patients. The study was approved by the Research Ethics Committee of Riga Stradins University (Approval No. 280426-6L, 29 April 2026).

### 2.2. Patient Data

For this cohort study, the sole inclusion criterion was undergoing LAA occlusion using the AtriClip PRO2 device. All patients meeting this criterion were included in the study.

Clinical, procedural, and follow-up data were obtained from electronic medical records. Baseline demographic characteristics, medical history, indication for LAA occlusion, antithrombotic therapy, echocardiographic findings, and procedural outcomes were collected.

Patients were followed during the index hospitalization and at a routine postoperative outpatient visit three months after the procedure. Three-month clinical follow-up data were available for all 14 patients (100%). In addition, rehospitalizations and major adverse clinical events documented in the electronic medical records were recorded from the index procedure through database closure on 1 July 2026. Imaging assessment of LAA closure was performed with intraoperative TEE according to clinical routine; however, there was no protocol-mandated, standardized CT or TEE imaging schedule for long-term assessment of closure durability.

Decisions regarding postoperative antithrombotic therapy were individualized. Discontinuation of oral anticoagulation was considered when complete LAA exclusion without residual flow or stump was confirmed intraoperatively by TEE. TEE findings were assessed in consultation with an echocardiography specialist, and the final decision regarding discontinuation of anticoagulation was made by the treating cardiac surgeon based on the intraoperative imaging findings and the individual clinical context.

Thromboembolic risk was assessed using the CHA_2_DS_2_-VASc score and bleeding risk using the HAS-BLED score. Surgical risk was estimated using EuroSCORE II and the Society of Thoracic Surgeons (STS) risk calculator. Procedural endpoints were reported separately and included thoracoscopic completion, conversion to sternotomy, complete LAA closure, and perioperative complications. Additional outcomes included mortality, stroke, rehospitalization, and antithrombotic therapy status during follow-up. Complete LAA closure was defined as absence of residual flow and residual stump on TEE assessment.

### 2.3. Procedural Technique

All procedures were performed under general anesthesia using a double-lumen endotracheal tube to facilitate selective right lung ventilation. Intraoperative transesophageal echocardiography (TEE) was used in all cases. Following thoracoscopic access to the left pleural cavity, carbon dioxide insufflation was established and the pericardium was opened below and parallel to the phrenic nerve. The AtriClip Pro2 device was positioned at the base of the LAA under TEE guidance with the aim of achieving complete appendage exclusion without a residual stump. After clip deployment, complete closure was confirmed intraoperatively by TEE. All patients were subsequently transferred to the cardiac surgical intensive care unit for postoperative monitoring.

### 2.4. Statistical Analysis

Given the small sample size, statistical analysis was descriptive. Continuous variables are presented as mean ± standard deviation (SD), range, and median with interquartile range (IQR), as appropriate; categorical variables are presented as frequencies and percentages. For key procedural and clinical proportions, 95% confidence intervals (CIs) were calculated using the Wilson method. No hypothesis testing or inferential comparisons were performed. Statistical analyses were performed using IBM SPSS Statistics software version 31.0.1.0 (IBM Corp., Armonk, NY, USA).

## 3. Results

### 3.1. Participant Characteristics

A total of 14 patients underwent standalone LAA occlusion during the study period. Mean age was 63.6 ± 16.7 years (range 22–79 years), and nine patients (64.3%) were female.

The cohort was clinically heterogeneous and included patients treated for different indications. Mean CHA_2_DS_2_-VASc score was 4.7 ± 2.0, and mean HAS-BLED score was 2.7 ± 1.1. Cerebrovascular disease was present in 11 patients (78.6%), including seven patients with a history of ischemic stroke, three with previous hemorrhagic stroke, and one with a transient ischemic attack.

Permanent atrial fibrillation was the most common arrhythmia subtype and was present in nine patients (64.3%). Additional baseline characteristics are summarized in Table 1.

### 3.2. Surgical and Clinical Outcomes

Thoracoscopic LAA occlusion was completed in 13/14 patients (92.9%; 95% CI 68.5–98.7%). One patient required conversion to median sternotomy because of intraoperative bleeding (1/14, 7.1%; 95% CI 1.3–31.5%).

Complete LAA occlusion without residual flow or stump was documented in all 14 patients (100%; 95% CI 78.5–100%). No repeat intervention for incomplete LAA closure was required.

Mean length of hospital stay was 10.6 ± 3.9 days (median 10.0; IQR 7.0–14.0). Perioperative complications occurred in 2/14 patients (14.3%; 95% CI 4.0–39.9%) and included bleeding requiring conversion to sternotomy and left-sided pneumonia. No perioperative stroke or mortality occurred (0/14 for each; 95% CI 0–21.5%). Mean EuroSCORE II predicted operative mortality was 2.10 ± 1.99% (median 1.56%; IQR 1.05–2.02%), and mean STS predicted operative mortality was 2.04 ± 1.11% (median 1.87%; IQR 1.07–2.44%).

During follow-up, the most frequently observed clinical events were heart failure-related rehospitalization (n = 2), post-cardiotomy pericarditis (n = 1), pneumonia with bilateral hydrothorax (n = 1), and pain requiring emergency department evaluation (n = 1).

Clinical outcomes are presented in Table 2.

### 3.3. Antithrombotic Therapy and Contraindications to Oral Anticoagulation

Before surgery, 13 patients (92.9%) were receiving oral anticoagulation, most commonly rivaroxaban (n = 8, 57.1%), followed by edoxaban (n = 4, 28.6%) and apixaban (n = 1, 7.1%). Two patients (14.3%) were receiving antiplatelet therapy, with one patient taking aspirin and one taking clopidogrel.

At hospital discharge, six patients (42.9%) were discharged without antithrombotic therapy. Six patients continued treatment with rivaroxaban, one patient received edoxaban, and one patient was prescribed a combination of apixaban and clopidogrel. At the three-month follow-up, clinical data were available for all 14 patients; antithrombotic therapy had been discontinued in 12 patients (85.7%; 95% CI 60.1–96.0%), while two patients (14.3%) remained on antithrombotic therapy.

The most common indication for LAA closure was a contraindication to long-term oral anticoagulation. The leading reasons were previous intracranial bleeding in five patients (35.7%), gastrointestinal bleeding in two patients (14.3%), and thromboembolic events despite anticoagulation in two patients (14.3%). Less common indications included macrohematuria, spontaneous hematomas during anticoagulation, and other bleeding-related complications.

Two patients had no contraindication to oral anticoagulation and underwent the procedure for other clinical reasons. Notably, one 22-year-old patient presented with ectopic atrial tachycardia originating from the LAA. Previous radiofrequency catheter ablation had been unsuccessful, and the patient subsequently developed progressive heart failure. Following thoracoscopic LAA closure, the arrhythmia resolved and left ventricular function improved. One patient underwent thoracoscopic LAA occlusion after a previous unsuccessful attempt at percutaneous LAA closure. Antithrombotic therapy and contraindications are presented in Table 3.

### 3.4. Echocardiographic and Functional Parameters

Complete LAA closure without residual flow or stump was documented in all 14 patients by intraoperative TEE. Postoperative CT or TEE was not routinely performed according to a standardized study protocol; therefore, systematic imaging data on long-term closure durability were not available.

At baseline, one patient was in NYHA functional class I, 11 patients were in class II, and two patients were in class III. At follow-up, four patients were in NYHA class I, eight in class II, and two remained in class III.

Mean left ventricular ejection fraction was 54.3 ± 13.6% before surgery and 50.1 ± 10.5% at follow-up. See Table 4.

## 4. Discussion

This study describes the initial single-center experience with standalone thoracoscopic LAA occlusion using the AtriClip Pro2 device in Latvia. Thoracoscopic completion was achieved in 13 of 14 patients, one patient required conversion to sternotomy, and complete LAA closure was documented in all patients. These observations should be interpreted in the context of an early feasibility series with a small sample size, heterogeneous clinical indications, and no comparator group.

Most patients had high thromboembolic risk and clinically important reasons to avoid long-term OAC, including previous intracranial or gastrointestinal bleeding and thromboembolic events despite anticoagulation. However, the cohort was heterogeneous: two patients did not have a conventional contraindication to OAC, and one 22-year-old patient underwent LAA exclusion for LAA-origin ectopic atrial tachycardia after unsuccessful catheter ablation. Pooling these distinct indications limits the interpretation and generalizability of aggregate clinical outcomes.

Complete LAA exclusion is an important technical endpoint. In our series, complete closure without residual flow or stump was documented in all patients, although one procedure required conversion to sternotomy. Wang et al. reported high procedural success in a substantially larger series of 144 thoracoscopic AtriClip Pro2 procedures [7]. Although our cohort is considerably smaller, the high rate of complete LAA exclusion observed in this initial experience is consistent with the technical outcomes reported in larger published series.

Complete LAA exclusion was confirmed intraoperatively by TEE in all patients. Postoperative CT or TEE was not routinely performed during follow-up, as imaging surveillance was based on clinical indications rather than a standardized study protocol. Consequently, the present study primarily reflects early clinical outcomes and does not provide systematic imaging data on long-term anatomic durability. Discontinuation of antithrombotic therapy during follow-up is therefore reported as an observed clinical management outcome.

Current guidelines recognize LAA management as an important component of stroke prevention in selected patients with AF [1]. Evidence supporting surgical LAA closure has been strengthened by LAAOS III, which demonstrated a reduction in ischemic stroke and systemic embolism when LAA occlusion was performed concomitantly with cardiac surgery [12]. In LAAOS III, patients underwent cardiac surgery for another indication and most continued anticoagulation after the procedure. Our study addresses a different clinical setting—standalone thoracoscopic epicardial LAA exclusion, predominantly in patients for whom long-term anticoagulation was unsuitable—and therefore provides complementary early clinical experience with this approach.

Percutaneous LAA occlusion using devices such as Watchman or Amulet represents an established alternative for stroke prevention in selected patients with AF [13]. Thoracoscopic AtriClip implantation provides epicardial exclusion of the LAA without leaving an intracardiac implant and may be particularly useful when a surgical approach is clinically appropriate or when percutaneous closure is not feasible. Recent evidence from the IMPRESSION-LAAC study further highlights the importance of achieving complete LAA exclusion. In patients undergoing Watchman FLX implantation, device compression was independently associated with the occurrence of peridevice leak at follow-up TEE [14]. Although the mechanisms of closure differ between endocardial devices and epicardial clipping, both approaches emphasize the importance of optimal device positioning and complete exclusion of the LAA. In our cohort, complete LAA exclusion without residual flow or stump was confirmed intraoperatively by TEE in all patients.

One patient in our series underwent successful thoracoscopic LAA exclusion after a previous unsuccessful percutaneous closure attempt. Although limited to a single case, this experience illustrates a potential role for epicardial LAA exclusion when percutaneous closure is unsuccessful or not feasible.

An additional and distinct indication for LAA exclusion in our cohort was LAA-origin ectopic atrial tachycardia. One 22-year-old patient had persistent ectopic atrial tachycardia originating from the LAA despite previous radiofrequency catheter ablation and subsequently developed progressive heart failure. Following thoracoscopic LAA exclusion, the arrhythmia resolved and left ventricular function improved. The LAA is a recognized, although uncommon, site of origin of focal atrial tachycardia, and its anatomical characteristics may make successful endocardial ablation challenging [8]. In selected patients with recurrent LAA-origin atrial tachycardia after unsuccessful catheter ablation, surgical exclusion of the appendage may therefore represent an alternative treatment strategy. Although this observation is limited to a single patient, the favorable clinical response in our case is consistent with the rationale for definitive exclusion of the arrhythmogenic LAA.

The present study reflects the early experience of a newly established thoracoscopic LAA occlusion program. The observed technical results demonstrate that the procedure was feasible at our center.

Several limitations should be acknowledged. This was a retrospective, single-center study with a small sample size, heterogeneous clinical indications, no control group, and relatively short follow-up. The cohort included two patients who underwent LAA exclusion for indications other than a contraindication to long-term OAC, including one young patient treated for LAA-origin atrial tachycardia, which should be considered when interpreting the overall outcomes. Although three-month clinical follow-up was complete, postoperative CT or TEE was not routinely performed according to a standardized study protocol, limiting assessment of long-term anatomic durability. The small sample size also results in wide confidence intervals for key outcomes and limits meaningful comparative analyses. Therefore, the findings should be regarded as early clinical experience demonstrating procedural feasibility, while larger prospective studies with standardized imaging and longer follow-up are needed to further evaluate safety and clinical efficacy.

Taken together, these findings demonstrate the technical feasibility of standalone thoracoscopic AtriClip Pro2 implantation and provide encouraging early clinical experience with this approach in Latvia. Further studies in larger patient populations with longer follow-up will help define its clinical outcomes and role in the management of selected patients requiring LAA exclusion.

## 5. Conclusions

Standalone thoracoscopic left atrial appendage occlusion using the AtriClip Pro2 device was successfully implemented at our cardiac surgery center in Latvia. In this initial 14-patient experience, thoracoscopic completion was achieved in 13 patients, with one conversion to sternotomy, while complete LAA exclusion was achieved in all patients. No perioperative stroke or mortality was observed, and antithrombotic therapy was discontinued in the majority of patients during early follow-up.

These findings demonstrate the technical feasibility of standalone thoracoscopic AtriClip Pro2 implantation and provide encouraging early clinical experience with this approach. Larger prospective studies with longer clinical follow-up and standardized imaging are warranted to further evaluate long-term closure durability and clinical outcomes and to better define the role of standalone thoracoscopic LAA exclusion in selected patients.

## Figures and Tables

**Table 1:** Characteristics of the population.

Number of patients, n	14
Female sex, n (%)	9 (64.3)
Age, years, mean ± SD (min–max); median (IQR)	63.6 ± 16.7 (22–79); 67.0 (54.8–76.8)
CHA_2_DS_2_-VASc score, points, mean ± SD (min–max)	4.7 ± 2.0 (1–7)
HAS-BLED score, points, mean ± SD (min–max)	2.7 ± 1.1 (0–4)
Mobility impairment:	
Yes, n (%)	7 (50.0)
Arrhythmia type, n (%):	
Paroxysmal atrial	3 (21.4)
fibrillation	
Persistent atrial fibrillation	1 (7.1)
Permanent atrial	9 (64.3)
fibrillation	
Atrial ectopic tachycardia	1 (7.1)
Cerebrovascular disease:	
Yes, n (%)	11 (78.6)
History of cerebral stroke, n (%)	10 (71.4)
Stroke type, n (%):	
Hemorrhagic stroke	3 (21.4)
Ischemic stroke	7 (50)
Transient ischemic attack	1 (7.1)

**Table 2:** Procedural characteristics and outcomes.

Length of hospital stay, days, mean ± SD (min–max); median (IQR)	10.6 ± 3.9 (6–18); 10.0 (7.0–14.0)
Procedural approach/endpoints, n (%): Thoracoscopic completion Conversion to sternotomy Complete LAA closure	13 (92.9) 1 (7.1) 14 (100)
EuroSCORE II operative mortality, %, mean ± SD (min–max); median (IQR) STS operative mortality, %, mean ± SD (min–max); median (IQR)	2.10 ± 1.99 (0.69–8.63); 1.56 (1.05–2.02) 2.04 ± 1.11 (0.94–4.68); 1.87 (1.07–2.44)
Perioperative complications, n (%): No complications Bleeding requiring conversion to sternotomy Left-sided pneumonia	12 (85.7) 1 (7.1) 1 (7.1)
Clinical events after discharge, n (%):	
Emergency department visit due to pain at the surgical site	1 (7.1)
Postcardiotomy pericarditis	1 (7.1)
Nosocomial pneumonia with bilateral hydrothorax and hyponatremia 3 days after discharge	1 (7.1)
Bradyarrhythmia requiring pacemaker implantation followed by MSSA infective endocarditis requiring pacemaker extraction	1 (7.1)
Rehospitalization due to heart failure decompensation	2 (14.3)

**Table 3:** Antithrombotic therapy and contraindications.

Anticoagulation therapy before operation, n (%): Yes	13 (92.9)
Type of anticoagulant used before operation, n (%): Rivaroxaban Apixaban Edoxaban	8 (57.1) 1 (7.1) 4 (28.6)
Antiplatelet therapy before operation, n (%): Yes	2 (14.3)
Type of antiplatelet therapy before operation, n (%): No Aspirin Clopidogrel	12 (85.7) 1 (7.1) 1 (7.1)
Antithrombotic therapy after discharge, n (%): No Rivaroxaban Edoxaban Apixaban + clopidogrel	6 (42.9) 6 (42.9) 1 (7.1) 1 (7.1)
Antithrombotic therapy 3 months after operation, n (%): No Yes	12 (85.7) 2 (14.3)
Clinical indications for LAA occlusion, n (%): Gastrointestinal bleeding Cerebral infarction while on anticoagulation Subarachnoid hemorrhage Macrohematuria on anticoagulation Spontaneous subcutaneous hematomas on anticoagulation Endometrial hyperplasia Multiple cerebral microhemorrhages Spontaneous intradural spinal hemorrhage (Th3–Th7) with paraplegia on OAC Traumatic subdural hematoma	Total 12 (85.7) 2 (14.3) 2 (14.3) 2 (14.3) 1 (7.1) 1 (7.1) 1 (7.1) 1 (7.1) 1 (7.1) 1 (7.1)

**Table 4:** Echocardiographic and functional parameters before and after surgery.

NYHA class before operation, n (%): NYHA I NYHA II NYHA III	1 (7.1) 11 (78.6) 2 (14.3)
Left ventricular ejection fraction before operation, %, mean ± SD (min–max); median (IQR)	54.3 ± 13.6 (24–68); 60.0 (55.8–60.0)
Complete left atrial appendage closure without residual flow or stump, n (%)	14 (100; 95% CI 78.5–100)
Left ventricular ejection fraction at follow-up, %, mean ± SD (min–max); median (IQR)	50.1 ± 10.5 (23–62); 54.5 (45.0–55.0) [available in 12/14 patients]
NYHA class after operation, n (%): NYHA I NYHA II NYHA III	4 (28.6) 8 (57.1) 2 (14.3)

## Data Availability

The data supporting the findings of this study are available from the corresponding author upon reasonable request.
